# OOPSIE (OOPS informatics extension)

**DOI:** 10.1093/bioadv/vbag278

**Published:** 2026-09-23

**Authors:** Stephanie B Telerman, Lajos Kalmar, Sandip K Patel, Anne E Willis, Kathryn S Lilley

**Affiliations:** Department of Biochemistry, University of Cambridge, Cambridge, CB2 1GA, United Kingdom; MRC Toxicology Unit, University of Cambridge, Cambridge, CB2 1QR, United Kingdom; MRC Toxicology Unit, University of Cambridge, Cambridge, CB2 1QR, United Kingdom; MRC Toxicology Unit, University of Cambridge, Cambridge, CB2 1QR, United Kingdom; Department of Biochemistry, University of Cambridge, Cambridge, CB2 1GA, United Kingdom

## Abstract

**Motivation:**

The RNA-bound proteome represents a dynamic and pleiotropic network of RNA-binding proteins (RBPs) that integrate diverse cellular pathways. RNA-binding dynamics profiling using cross-linking–based enrichment strategies including Orthogonal Organic Phase Separation (OOPS), generates complex peptide-level quantitative mass spectrometry datasets, yet dedicated computational workflows for standardized normalization and differential analysis remain limited.

**Results:**

OOPS relies on phase partitioning of RNA binding proteomes, upon UV cross-linking. As glycoproteins have been shown to co-partition independently of cross-linking due to their physicochemical properties, they provide a cross-link-independent reference population that can be leveraged for normalization of RNA-binding-dependent changes. We introduce OOPSIE (Orthogonal Organic Phase Separation Informatics Extension), an R-based script that provides a reproducible framework of peptide-level OOPS-derived data using protein-level normalization and differential analysis of RNA-binding capacity. We demonstrate its utility using OOPS-derived datasets generated from A549 lung adenocarcinoma cells.

**Availability and implementation:**

OOPSIE is implemented in R as a standalone workflow and is freely available at https://github.com/CambridgeCentreForProteomics/oopsie, together with documentation and example datasets.

## 1 Introduction

RNA-binding proteins (RBPs) are central regulators of post-transcriptional gene expression and are increasingly profiled across diverse biological systems and disease contexts (Gerstberger *et al*. 2014, Harvey *et al.* 2017, Hentze *et al.* 2018, Kang *et al*. 2020, Quintas *et al.* 2024, Turner and Petkau 2026). Cross-linking–based enrichment strategies have been developed to capture these RNA-RBP interactions, including approaches such as RNA interactome capture (RIC) (Perez-Perri *et al.* 2021), Phenol-Toluol extraction (PTex) (Urdaneta *et al.* 2019), protein-crosslinked RNA extraction (XRNAX) (Trendel *et al.* 2019), total RNA-associated protein purification (TRAPP) (Shchepachev *et al.* 2019), or Orthogonal Organic Phase Separation (OOPS) (Queiroz *et al.* 2019), which enable global capture dynamics of RNA-binding capacity of RBPs from quantitative mass spectrometry (MS) datasets (Villanueva *et al.* 2020, 2024, Monti *et al.* 2024). However, downstream computational analysis remains fragmented, with limited standardized frameworks for normalization, differential RNA binding modelling, and functional interpretation.

Here, we present OOPSIE, an R-based computational workflow that standardizes quantitative RBP-RNA binding analysis from OOPS-derived MS data. OOPSIE integrates peptide-level pre-processing with a biologically informed glycoprotein-based normalization strategy to correct cross-linking-dependent abundance shifts, annotation, and enrichment analysis within a modular and reproducible pipeline.

We applied OOPSIE to OOPS-derived mass spectrometry data from A549 lung adenocarcinoma cells to assess differential RNA-binding between cross-linked and non-cross-linked samples. The workflow enables a robust identification of differentially binding capacity RBPs, and downstream Gene Ontology enrichment analysis.

## 2 Methods

OOPSIE is implemented in R (using version 4.5) and distributed as a standalone R script. It operates on peptide-level quantitative mass spectrometry intensity tables and supports both data-dependent acquisition (DDA) and data-independent acquisition (DIA)-derived quantification tables.

The workflow is modular and reproducible, integrating peptide preprocessing, protein-level summarization, normalization, differential occupancy modelling and functional interpretation within a unified framework.

### 2.1 Data import and preprocessing

Peptide group quantification tables are imported directly into R as data frames, from which sample-specific abundances are extracted to construct the quantitative matrix used for downstream analyses. Contaminant proteins are removed using the cRAP (Common Repository of Adventitious Proteins) database (Mellacheruvu *et al.* 2013). Valid UniProt accession numbers (Bateman *et al.* 2023) are retained via the “Master.Protein.Accessions” column, and only uniquely assigned peptides are included for downstream analysis. Peptide intensities are log2-transformed to stabilize variance and improve interpretability.

### 2.2 Data structuring

Quantitative data are stored in a QFeatures object (Gatto and Vanderaa 2026), which integrates the peptide intensity matrix with feature metadata (peptide sequences and associated protein identifiers) and sample metadata (experimental group annotations, including cross-linked and non-cross-linked samples and replicates). This structure ensures compatibility with Bioconductor workflows and supports transparent, reproducible preprocessing.

### 2.3 Data quality assessment and peptide filtering

Quality control includes visualization of peptide abundance using density plots of log2-transformed intensities and missingness patterns to identify shifts or technical factors. Peptides with customizable missingness ratios are removed and only proteins supported by at least two quantified peptides are retained.

### 2.4 Protein-level aggregation

Filtered peptide intensities are aggregated to the protein level using UniProt identifiers (Master.Protein.Accessions). Robust median-based summarization is used to reduce the influence of outliers. Missing value distributions are reassessed post-aggregation to confirm improved data completeness and identify potential further technical issues.

### 2.5 Functional annotation

Proteins are annotated by matching their identifiers, supplementing the data table with more easily recognizable gene names while retaining UniProt accession numbers as the unique identifiers. The OOPS method relies on the physicochemical partitioning of macromolecules during phase separation. As both glycans and RNA are hydrophilic polymers, glycoproteins and RNA-protein adducts cannot be distinguished in the interface (Queiroz *et al.* 2019). For subsequent data processing purposes, we annotate both known glycoproteins using a curated species-specific reference list and RBPs using the Gene Ontology term GO:0003723 (RNA binding) (Carbon *et al.* 2021). Functional annotations are incorporated into the feature metadata of the protein-level QFeatures object.

### 2.6 Normalization and multivariate analysis

Principal Component Analysis (PCA) is applied to assess sample clustering and variance structure. To correct cross-linking–dependent abundance shifts and standard measurement errors (Callister *et al.* 2006), OOPSIE implements a biologically informed glycoprotein-based normalization strategy. During this normalization process we only use the glycoproteins (instead of the full proteome set) to perform a standard log-median normalization and correct all protein abundances with the calculated normalization factors. Furthermore, glycoproteins serve as an internal reference class, as their partitioning behaviour is expected to be cross-link-independent (Queiroz *et al.* 2019). A robust linear model (M-estimation; MASS::rlm) (Venables and Ripley 2002) is fitted using averaged log-transformed glycoprotein abundances only, comparing the two groups, cross-linked vs non-cross-linked samples. This regression defines the expected relationship between conditions under the null assumption of unchanged occupancy. To account for abundance-dependent variance, fit is modelled as a function of abundance using LOESS (local polynomial regression fitting) (Cleveland and Devlin 1988) on the glycoprotein set. The resulting variance estimates are used to construct abundance-level–dependent 95% prediction intervals around the fitted regression line.

Proteins falling outside these prediction intervals are classified as significantly enriched or depleted in the cross-linked condition compared to the non-cross-linked condition (*P* < .05), representing differential occupancy events. This approach generates statistical boundaries directly from the empirically stable glycoprotein reference population. From the fitted regression model, enrichment fold-change scores are computed as the deviation of each protein from the expected abundance relationship between conditions. These scores enable quantitative comparison between known RBPs from the literature and newly identified RBP candidates.

## 3 Results

OOPSIE (Fig. 1A) was applied to OOPS-derived quantitative mass spectrometry data from human A549 lung adenocarcinoma cells comparing cross-linked and non-cross-linked samples (*n* = 5 per condition). Principal component analysis of the filtered protein abundance matrix demonstrated clear separation between cross-linked and non-cross-linked conditions (Fig. 1B). Glycoprotein-based normalization corrected cross-linking–dependent abundance shifts. Differential occupancy analysis identified proteins exhibiting significant cross-link–dependent changes (Fig. 1C). This included both previously annotated RNA-binding proteins and candidate novel RBPs, with the distribution of enrichment intensities showing substantial overlap between known and novel RBPs (Fig. 1D), supporting the robustness of the detection strategy. Subsequent Gene Ontology (Yu *et al.* 2012) molecular function enrichment analysis of significant proteins revealed functional categories consistent with RNA binding and post-transcriptional regulation (Fig. 1E), supporting the biological validity of the detected occupancy changes.

**Figure 1:**
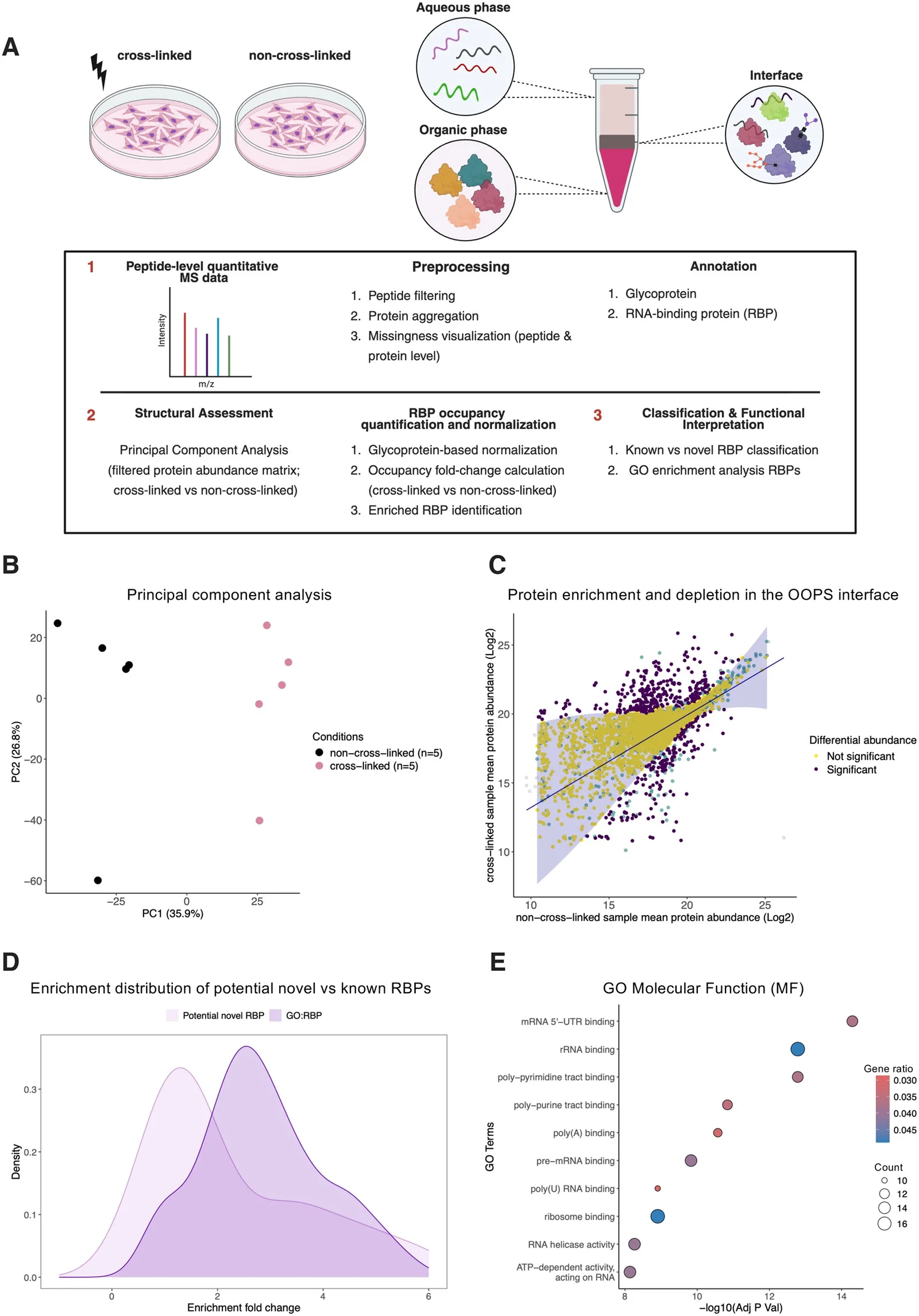
Overview and application of OOPSIE. (A) Schematic representation of the OOPSIE workflow, including the cross-linking of the cell samples and the phases extraction, preprocessing of peptide-level quantitative MS data, RBP occupancy quantification and normalization, and functional interpretation. (B) Principal component analysis (PCA) of filtered protein abundance matrices from A549 lung adenocarcinoma cells (cross-linked versus non-cross-linked; n = 5 per condition). (C) Relationship between cross-linked and non-cross-linked average protein abundances. Glycoproteins are coloured light blue, non-glycoproteins with significant (dark purple) or not significant (yellow) occupancy change are highlighted, abundance-dependent 95% prediction interval is plotted as a transparent blue ribbon. (D) Distribution of enrichment fold-change values for potentially novel and previously known RBPs (GO:RBP, annotated with GO:0003723). (E) Gene Ontology molecular function enrichment analysis of proteins with significant positive enrichment (FC > 0). The figure was created with BioRender.com.

Differential RNA-binding occupancy analysis between conditions (e.g. treatment vs control, cell state) can be performed by correlating the enrichment fold-change values generated by the script (the pipeline needs to be executed separately for each condition). In addition, by combining OOPS data with data generated for the total cellular proteome, changes in overall protein abundance can be distinguished from changes in RNA binding capacity of RBPs between conditions.

## 4 Conclusion

OOPSIE is an open-source framework for standardized analysis of OOPS-derived quantitative proteomics data. By using glycoproteins as a cross-link-independent internal reference population, it enables normalization and identification of RNA-binding occupancy changes, supporting discovery of both established and candidate RBPs. The script generates analysis-ready figures and summary tables to support interpretation. While demonstrated here using A549 lung adenocarcinoma datasets, OOPSIE is applicable across diverse experimental systems, species, and comparative designs and provides a reproducible approach for studying RNA-binding dynamics.

## Data Availability

The data underlying this article are available in Apollo (institutional repository of the University of Cambridge) at https://doi.org/10.17863/CAM.135464.
